# Supplementary material for: Validation of a Temperature-Feedback Controlled Automated Magnetic Hyperthermia Therapy Device
Source: Cancers (Basel). 2023 Jan 4;15(2):327. doi: 10.3390/cancers15020327 (PMC9856953; doi:10.3390/cancers15020327)
Supplement: Supplementary file 1 [file cancers-15-00327-s001.zip › cancers-2060303-supplementary.pdf]

SUPPLEMENTARY MATERIALS

Validation of a temperature-feedback controlled automated magnetic hyperthermia therapy device

Anirudh Sharma[1*], Avesh Jangam[2*], Julian Yung Shen Low[2], Aiman Ahmed[2], Nageshwar Arepally[2], Benjamin Rodriguez[3,4], Joseph Borrello[3,4], Alexandros Bouras[4,5], Lawrence Kleinberg[1], Kai Ding[1,6], Constantinos Hadjipanayis[4,5], Dara. L. Kraitchman[7], Robert Ivkov[1,6,8,9#], Anilchandra Attaluri[2#].

Text S1. Safety temperature control design

To meet device safety requirements listed in section 2.3, the in-house designed LabVIEW (National Instruments, Austin, TX) program included user-defined safety threshold temperatures, $T_{threshold}$ (eg. 50 °C), which if exceeded by the measured temperature at the sensor, ($T > T_{threshold}$), triggered a 0 V analog signal to power down the AMF. This signal immediately lowers the AMF to 0 kA/m in the coil (controller feedback voltage 0 V), until the temperature at the probe satisfied $T < T_{threshold}$. A second safety temperature probe was connected to a separate dedicated FISO TMI4 temperature conditioner (FISO, Quebec) to record the temperature, independent of the controller, as a risk management requirement (in case of feedback sensor damage). This probe, in the clinical setting, would be placed strategically by the user (eg. subcutaneous near the skull or rectal temperature) to monitor physiological or tissue surface temperatures during a MHT treatment. The algorithm is designed to drop the power to the specified lower bound (e.g., 0 V) within 0.2 s if either of the safety thresholds in either of the probes (feedback sensor or safety sensor) are exceeded and subsequently, recover the power for temperature control once the measured temperature at both locations drop to within the user-specified safety limits. Additionally, the upper and lower power supply bounds (0-5 V range corresponding to 0-100% power), which are used to define the AMF amplitude range, are specified in the program by the user based on performance and safety considerations (eddy current heating, thermal runaway, electrical safety, and power tripping prevention). 0.25-1.6 V (4.2-12 kA/m peak at 160 kHz) controller analog output range complied with these safety requirements while meeting heating performance requirements for experiments conducted in

this study. Finally, the power supply has a manual override emergency stop push-button to turn the power off in case of controller failure or during an emergency.

SI Figs S2A shows the power-limiting overriding characteristic for the safety sensor. When the temperature at this sensor location (blue solid curve) exceeds the safety threshold temperature, $T_{threshold,}$ eg. 27 °C (blue horizontal dashed line, $T > T_{threshold}$), a 0 V analog signal from the controller is sent to the power supply, as observed by the sharp drop in controller voltage (red curve). This signal immediately lowers the AMF to 0 kA/m in the coil, until measured temperature $T < T_{threshold}$. As a result, during this AMF OFF period, the temperature at the feedback-sensor location (black curve) starts dropping because of sample/treatment-region cooling. Once the safety temperature (blue curve) decreases back to below the threshold (blue dashed line, $T < T_{threshold}$), the power and PID function are restored, and the controller tracks the temperature at the feedback sensor-location to the reference setpoint temperature (green dashed line, 25 °C). It is worth noting that the PID function is programmed as a lower priority than the safety condition, and therefore, is always overridden by the safety sensor condition (AMF ON if $T < T_{threshold}$, AMF OFF if , $T > T_{threshold}$). A similar user-defined threshold is set for the measurement/feedback temperature sensor to limit the power based on the maximum tolerable upper limit in the treatment-region (e.g., tumor margin). SI Fig S2B demonstrates this feature in a situation where the PID controller is untuned, and temperature oscillations at the feedback sensor location crosses the user-defined safety limit (green dashed line, 30 °C). These two results in SI Fig S2 validate the controller's priority to safety over temperature regulation.

SUPPLEMENTARY FIGURES

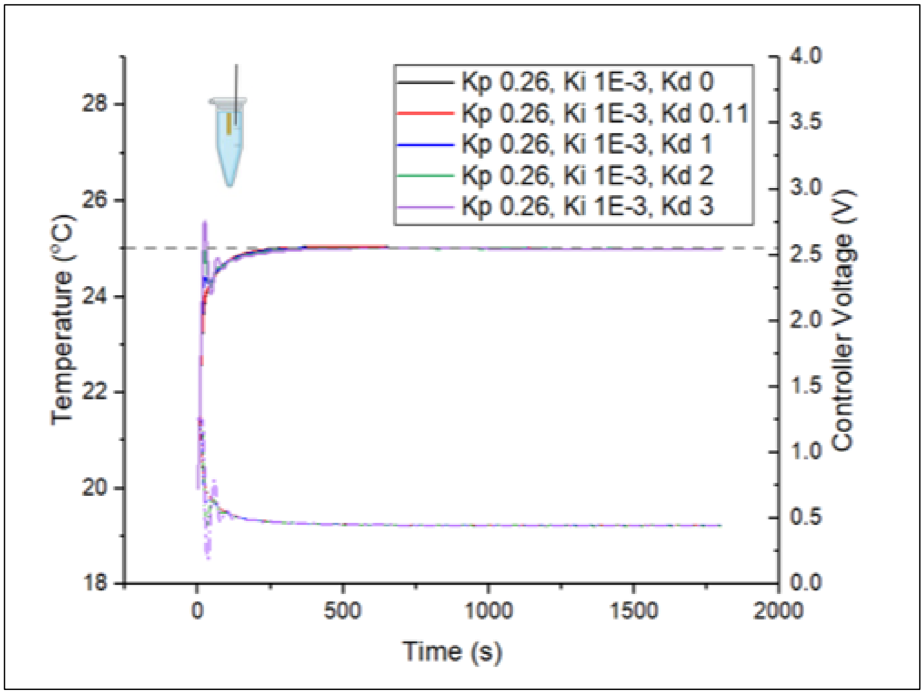

Figure S1. Simulated temperature vs time responses for the agarose gel + Cu wire system (Fig 2), with temperature feedback control at the sensor location (1.3 mm from Cu heat source surface), as a function of various PID gain combinations (K_p , K_i , K_d). The setpoint temperature (T_{ref}) is 25ºC (dashed green line). Responses are measured to a parameter sweep of (K_p , K_i , K_d) in the neighborhood of calculated gains (*0.,26*, *0.001*, *0.11*) (Section 2.4) to determine gains that would result in a shortest rise time, shortest settling time and overshoot ≤1ºC. In the experimental system, a K_i of 0.001 $s^{-1}K^{-1}$ was too high to prevent integral windup and consequent temperature and power oscillations, which the simulations do not capture. In the experimental setup, the windup effect of the integral gain was lowered by reducing K_i to 0.0001 $s^{-1}K^{-1}$ (Fig 2). Increasing K_i to reduce the rise time, therefore, was not feasible to achieve a critically damped system. Increasing the derivative gain, K_d, from 0.11 to 3 allowed reduction in rise time and better control over the overshoot past the setpoint temperature, providing a better alternative to the slower response simulated with calculated gains (*0.,26*, *0.001*, *0.11*). The lower signals on the plot show the controller voltage (right y-axis) vs time for the gain combinations tested.

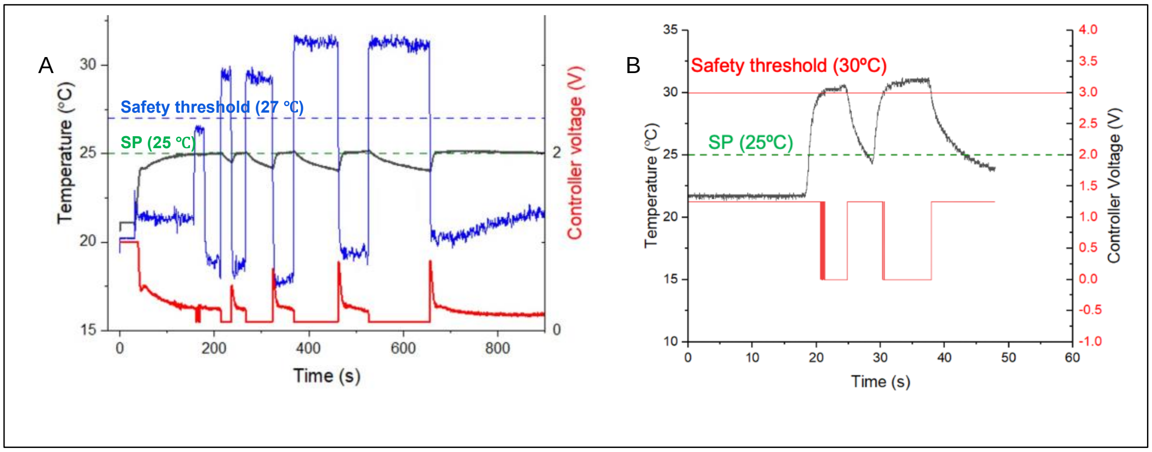

Figure S2. Safety and reliability of the temperature controller. (**A**) Temperature (left y-axis) and controller voltage (right y-axis) vs time responses during the safety testing of the temperature controller. To prevent thermal runaway, the LabVIEW code was designed to include a user-defined safety threshold temperature, $T_{threshold}$, eg. 27 °C (blue horizontal line), which if transgressed by the temperature sensor would trigger a 0 V analog signal from the controller to the power supply, as observed by the sharp drop in controller voltage to 0 V (red curve). This signal immediately lowers the AMF to 0 kA/m in the coil, until measured temperature $T < T_{threshold}$. A natural consequence of this 0 W power input to the coil is that the temperature at the feedback-sensor location (black curve) also starts dropping because of sample cooling. Once the safety temperature drops below the threshold (blue line), the power and PID function is restored, so the temperature at the sensor-location increases back to the setpoint temperature (green dashed line, 25 °C). The safety probe is connected to an independent temperature reader (FISO TMI4), different from the FISO SPC-HR and temperature controller, to prevent safety temperature readout failure in case of controller failure. (**B**) Temperature (left y-axis) and controller voltage (right y-axis) vs time responses during the safety testing of the temperature controller demonstrates the power limiting feature when the feedback temperature sensor transgresses a safety threshold. In this case, the transgression of the user-defined safety threshold temperature, $T_{threshold}$, (red horizontal line, 30 °C), triggers a 0 V analog signal from the controller to the power supply, as observed by the sharp drop in controller voltage to 0 V. This signal immediately lowers the AMF to 0 kA/m in the coil, until measured temperature $T < T_{threshold}$.

This may occur when the PID controller is improperly tuned, as shown in the figure. The safety threshold in this case prevents ablation or run-away at the feedback sensor location. In addition, the power supply has a manual override emergency stop button to manually shut the power off in case of controller failure or an emergency.

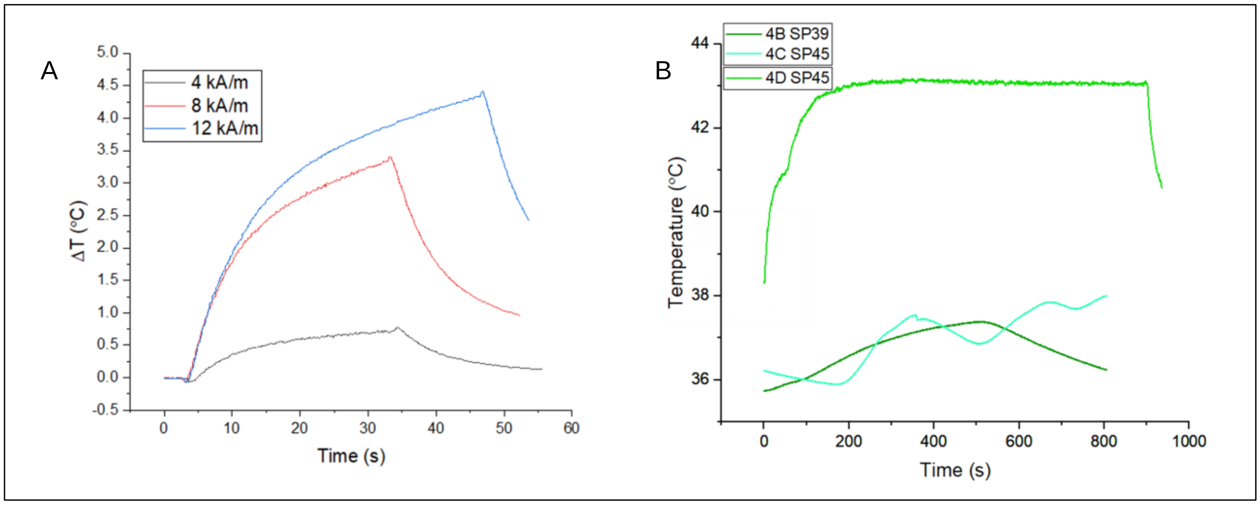

Figure S3. Supporting temperature vs time data for *in vivo* validation of the MHT temperature controller in a canine research subject. (**A**) Increase in temperature (ΔT, °C) at the catheter's MNP port-tip location in the canine brain, for AMF pulses of different amplitudes (4, 8 and 12 kA/m peak, 160 kHz). The AMF ON time for each pulse was at least 30 s. The rates of increase in temperatures from AMF pulses, previous *ex vivo* experiments, and *in vivo* real time temperature responses were used to estimate and fine tune PID gains (K_p , K_i , K_d) to *(2, 1E-4, 2.5)*, for an optimal controller response (Fig 4D). (**B**) Temperature vs time recorded from a fiber optic temperature probe inserted subcutaneously in the canine head (S, green in Fig 4A) to monitor the temperature increase from eddy current heating during the AMF treatments in 4(B)-(D), respectively. Due to uncertainty in the placement of this specific temperature sensor relative to the infused MNPs in the canine head, the effect of eddy current heating is confounded. This sensor was used as a "safety" sensor, with an upper cutoff limit set at 45℃ to prevent healthy tissue ablation. The maximum temperature recorded at this sensor location was ~ 43℃ for the AMF treatment corresponding to Fig 4D.

SUPPLEMENTARY TABLES

Table S1. Cu wire dimensions used for experimental verification of gel + Cu wire system.

Materials	Radius	Length	Weight
**Cu wire**	1 mm	4.52 mm	0.104 g

Table S2. Material properties used in COMSOL simulations for PID temperature feedback control

Parameters	Mean	Uncertainty
Magnetic Field Amplitude (peak)	9.8 kA/m	±5%
Gel density (rhogel)	960 kg/m^3	±1%
Specific Heat (Cpgel)	3900 J/(kg.K)	±1%
Thermal Conductivity (kgel)	0.566 W/m.K	±1%
Probe distance (dist)	1.3 mm	±5%
Electrical Conductivity (sigmagel)	2.1 (S/m)	±5%
Convective heat transfer coefficient (h_{conv})	21 (W/m^2.K)	±5%

Table S3. Parameter list for calculating preliminary PID gains

Parameter	Value
τ_1	2.96 s
τ_{63}	252.0 s
τ_2	249.04 s
**g**	95.83 K/V (23/0.24)
ω_n	0.2 rad/s
ζ	1
τ_d	2.17 s
κ	1E-3 s^{-1}K^{-1}
K_p	0.26 K^{-1}
K_i	1E-3 s^{-1}K^{-1}
K_d	0.11 s.K^{-1}
u_{ctrl}	0.24 V
T_∞	293.15 K

Table S4. NIST Traceable Cu wire dimensions used for *ex vivo* validation trials

Materials	Radius (mm)	Length (cm)	Width (cm)	Thickness (cm)	Weight (g)
Cu wire 1	1	0.5	x	x	0.116
Cu wire 2	1	0.65	x	x	0.153
Cu wire 3	1	0.6	x	x	0.135
Cow liver	x	15	13	3	497.5
